# Creating beauty: creativity compensates for low physical attractiveness when individuals assess the attractiveness of social and romantic partners

**DOI:** 10.1098/rsos.160955

**Published:** 2017-04-19

**Authors:** Christopher D. Watkins

**Affiliations:** Division of Psychology, School of Social and Health Sciences, Abertay University, Dundee, Scotland DD1 1HG, UK

**Keywords:** creativity, mate choice, allies, story-telling, sexual selection

## Abstract

Although creativity is attractive in a potential mate, it is unclear (i) whether the effects of creativity on attractiveness generalize to other social contexts and (ii) whether creativity has equivalent effects on men's and women's attractiveness. As social knowledge of creativity may either enhance or ‘offset’ the appeal of social partners who differ in physical attractiveness, three repeated measures experiments were conducted to directly address these issues. Here, participants rated a series of face–text pairs for attractiveness on trials that differed in one of four combinations of facial attractiveness (attractive and less attractive) and creativity (creative and less creative), rating story-tellers in two experiments (short interpretations of an identical painting) and creative ideas in a further experiment (alternative uses for an everyday object). Regardless of the sex of the judge, creativity and facial attractiveness had independent effects on men's overall attractiveness (initial experiment) and, in further experiments, more substantial effects on the attractiveness of men with less attractive faces than men with attractive faces (when using a different measure of creativity) and specific effects on the attractiveness of individuals with less attractive faces (when using different face stimuli). Collectively, across three experiments, these findings suggest that creativity may compensate for putative cues to lower biological ‘quality’ and that the benefits of creativity to social groups more generally enhance attraction to creative men (in two experiments) and creative men and women (one experiment). More broadly, the data suggest that species can integrate knowledge of cognitive intelligence with visual cues to biological ‘quality’ to facilitate mate and/or ally choice.

## 1. Introduction

Cognitive intelligence is an attractive trait in a mate if it displays low exposure to environmental stressors and/or an ability to provide benefits that increase the reproductive fitness of partner or offspring (reviewed in [1]). Indeed, intelligence may have been shaped, at least in part, by sexual selection [2]. Consistent with this proposal, cognitively complex skills such as song repertoire are positively correlated with reproductive success in birds (reviewed in [3]). Moreover, other complex skills such as vocal mimicry during courtship [4] and problem-solving ability are correlated with mating success in birds [5]. Innovative or novel displays may present conspecifics with a source of valuable information to transmit between groups more generally in specific contexts, such as that demonstrated in the transmission of novel whale song across groups ([6,7]; reviewed in [8]). Collectively, creativity, as an index of cognitive intelligence (e.g. [1,8–11]), may be a particularly desirable trait in a mate or social partner.

In humans, creativity and intelligence are attractive in a romantic partner (reviewed in [12]), with recent evidence in female twins suggesting that preferences for these traits have a heritable basis [13]. Moreover, among creative professionals, dimensions of schizotypy (i.e. potentially costly traits) such as cognitive aberrations and magical thinking have an indirect effect on their mating success via the extent of their self-reported creative activity [14]. As creative displays are thought to have particular benefits to the reproductive fitness of those who compete for the more-selective sex (e.g. [15]), most research on creativity and mate choice has focused on the attractiveness of creativity in men (e.g. [11,16]), although women's attractiveness may also be enhanced via their creativity (see [12] for discussion). Consistent with this proposal, experimentally activating both short- and long-term mating goals directly increases men's creativity, while mating goals increase women's creativity in long-term contexts only [12]. Collectively, both men and women may enhance their attractiveness through creative displays.

Although creativity may be a desirable quality in humans and non-human species (e.g. [1,8,12]), it is unclear whether social knowledge of one's creativity has effects on attractiveness that are independent of, or are qualified by, physical indices of biological quality such as facial attractiveness ([17]; see also [18]). Indeed, studies testing for integration of social knowledge with physical cues on social judgements of attractiveness are novel (see [19] for an exception) and, in non-human species, no empirical work, to the author's knowledge, has directly tested whether simultaneous assessment of attractiveness and putative cues to intelligence shapes mate choice (reviewed in [1]). While previous work suggests that videos of creative men are judged as attractive by women when statistically controlling for differences in men's attractiveness [11], this work did not examine written creative output or consider whether their findings generalize to creative displays by women or contexts unrelated to mating, such as the attractiveness of a social partner more generally. Here, this issue is addressed using both faces and short-story extracts that, unknown to participants, have been rated for attractiveness and creativity, respectively, in order to test for effects of both story-telling ability and facial attractiveness on global attractiveness.

As creative displays are thought to provide men with a particular advantage in mating success (e.g. [9,11,15,16]), creativity would be predicted to have an *independent* effect on men's but not necessarily women's attractiveness if, for example, *physical* attractiveness has a stronger positive effect on women's than men's mating success (e.g. [20]; see also [21]), which in turn, reduces (or eliminates) any positive effects of creativity on their *attractiveness* when integrated with knowledge of their physical attractiveness. However, if the effects of creativity on attractiveness are qualified by facial attractiveness, we can establish whether creativity enhances attractiveness to a greater extent when individuals judge less attractive faces (i.e. cognitive intelligence ‘offsets’ low physical attractiveness) or, alternatively, when individuals judge more attractive faces (i.e. social knowledge of creativity enhances the appeal of physically attractive social/romantic partners). Evidence that creativity has effects on attractiveness that are independent of, or interact with, facial attractiveness, would provide initial experimental evidence in light of theoretical discussion on the role of cues to biological quality versus cues to cognitive intelligence on reproductive fitness [1].

Finally, the experiment will test whether creativity enhances attractiveness to a greater extent when judging opposite-sex faces (i.e. potential mates) or if the effects of creativity on attractiveness generalize across both mating and non-mating contexts. While evidence for the latter prediction would not rule out the utility of creativity in mate choice, it would be consistent with recent proposals that innovation is important in the transmission of valuable information across groups more generally [8]. Indeed, attraction to written creativity may generalize to non-mating contexts if, for example, language transmits useful information at no loss to the transmitter [22], and intelligence, a prestigious trait [23], is important for increasing the leverage of group members [24] and for group cooperation and cohesion [25] which, in turn, can facilitate access to mates (e.g. [26]).

## 2. Method

### 2.1. Participants

Eighty-nine participants (21 male, *M*_age_ = 23.01 years, s.d. = 8.18 years) took part in the experiment. Participants took part in class under test conditions, in partial fulfilment of course credit. The experiment was presented alongside other randomized tasks unrelated to the current research.

### 2.2. Face stimuli

A publically available database of face photographs (KDEF, [27]), which had been independently rated for attractiveness by a panel of judges, was used in the current experiment (standardized attractiveness and intelligence ratings provided by Oosterhof & Todorov [28]). Within the face set, eight male and eight female faces were selected, with four attractive faces selected for each sex and four faces around the mid-point of the entire set selected for each sex. All photographs were of Caucasian individuals taken in a standardized manner with neutral expression, direct gaze and no adornments (562 × 762 pixels). The mean attractiveness rating of the attractive faces (*M*_men_ = 0.98, *M*_women_ = 1.04) was greater than the mean attractiveness rating of the less attractive faces (*M*_men_ = −0.35, *M*_women_ = −0.36, *t*_14_ = 23.22; *p* < 0.001). Within each face set, male and female faces did not differ from one another on rated attractiveness (both *t*_6_ < 0.56; both *p* > 0.60). When split by gender, the attractive face sets did not differ in rated intelligence from the less attractive face sets (both absolute *t*_6_ < 1.51; both *p* > 0.18). The attractive men did not differ in rated intelligence from the attractive women and the less attractive men did not differ in rated intelligence from the less attractive women (both absolute *t*_6_ < 1.53; both *p* > 0.18).

### 2.3. Story extracts

**Figure 1.**
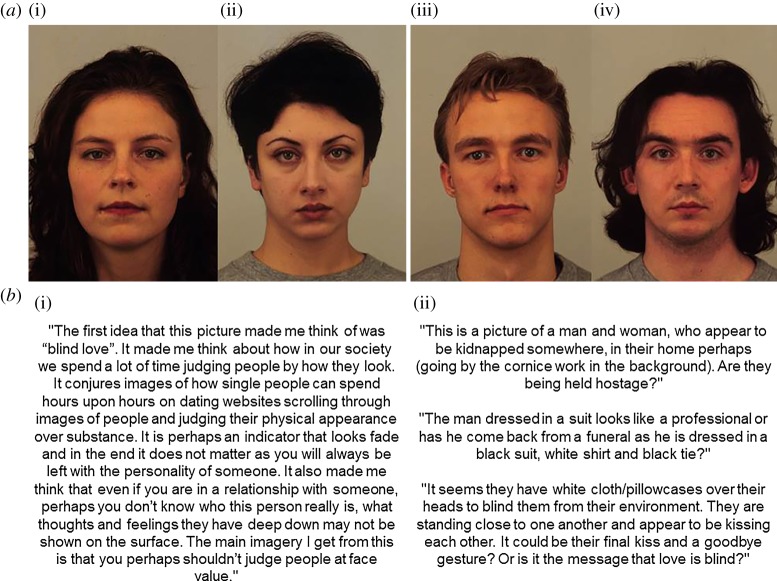
Example attractive (*a*(i)(iii)) and less attractive ((*a*(ii)(iv))) male and female faces. Example short-story extracts rated as creative (*b*(i)) and less creative (*b*(ii)). Extracts and faces were matched by sex in the experiment.

A separate group of 38 participants (18 male, *M*_age_ = 27.76 years, s.d. = 7.01 years) provided short-story extracts in the laboratory prior to the experiment. Using procedures adapted from Griskevicius *et al*. [12], participants were given up to 5 min to write a short story based on what they thought was happening in a painting (an A3 landscape colour printout of *The Lovers* by René Magritte, 1928). This painting was selected specifically in order to measure creativity in the context of romantic writing. Participants were not encouraged to be creative or informed that we were measuring their creativity. Each extract (Mean length = 105 words, Mean time to completion = 285 s) was then proofread by the researcher for mistakes with spelling or grammar and then rated by a panel of judges (three male, three female, *M*_age_ = 26.78 years, s.d. = 2.85 years) for eight traits related to creativity (creative, original, clever, imaginative, captivating, funny, entertaining and charming) on a 1 (not at all) to 9 (very) scale (*sensu* [12]). Raters were explicitly informed that each extract was produced by a separate individual looking at an identical painting and that each individual was given the same amount of time to write a short story based on what they thought was happening in the image. Raters were not shown the painting nor were they given any information about the actual painting. Agreement between judges was acceptable (Cronbach's α = 0.72). Two judges (one male and one female) only rated a subset of extracts; however, all data are included in correlational analyses. All of the dimensions related to creativity were significantly correlated with one another (all *r*_38_ > 0.47, all *p* < 0.01), except for ‘funny’ and some of the rated dimensions (notably ‘creative’ *r*_38_ = 0.11, *p* = 0.52). As the focus of the current experiment was creativity, a composite measure of creativity was created by averaging all of the ratings for each extract except its rated funniness (*M*_global creativity_ = 3.74, s.d. = 0.78). From these ratings, eight extracts produced by men (*M*_high male_ = 4.84, s.d. = 0.10, *M*_low male_ = 3.34, s.d. = 0.39) and eight extracts produced by women (*M*_high female_ = 4.80, s.d. = 0.56, *M*_low female_ = 3.34, s.d. = 0.16) were selected. To measure spontaneous creativity, extracts were only selected if the participant reported after the task that they were not familiar with the specific painting. Half of the extracts were creative and half were less creative, with extracts from the two sets differing significantly from one another on global rated creativity excluding funniness (*t*_14_ = 9.08; *p* < 0.001). Within each set, extracts did not differ in creativity according to the gender of the writer (both *t*_6_ < 0.12, both *p* > 0.91). Extracts were then randomly paired with faces by the experimenter, resulting in (separately for each gender) two attractive–creative face-extract pairs, two attractive–less-creative face-extract pairs, two less-attractive–creative face-extract pairs and two less-attractive–less-creative face-extract pairs (see examples in figure 1). Extracts and faces were matched by sex. All participants rated the same face-extract pairings.

### 2.4. Procedure

Participants were informed that the task involves making judgements about individuals, and were explicitly informed that each of the 16 pictured individuals in the experiment were provided with the same painting and the same amount of time to write a short story based on what they thought was happening in the image. They were instructed that, for each individual they see, they should read their extract and rate how attractive they judge each person to be. Participants were not shown the painting nor were they provided any information about the actual painting. The task was run via surveymonkey.com. Each facial photograph was centred on the screen with the short-story extract centred below the picture (eight male trials and eight female trials). Participants were asked to indicate how attractive they thought the individual was using the scale: much less attractive than average (=1), less attractive than average (=2), slightly less attractive than average (=3), of average attractiveness (=4), slightly more attractive than average (=5), more attractive than average (=6), much more attractive than average (=7). Trial order was randomized.

### 2.5. Initial processing of data

Data for each participant consist of their mean response across two trials to each combination of facial attractiveness (attractive and less attractive) and creativity of extract (high and low). This repeated measures data were calculated separately for judgements of men and judgements of women. High scores indicate high rated attractiveness based on integration of knowledge of their facial attractiveness with knowledge of their creativity.

## 3. Results

A mixed-design ANOVA with the within-subjects factors *face sex* (male and female), *facial attractiveness* (high and low) and *creativity* (high and low) and the between-subjects factor *sex of participant* (men and women) was conducted. This analysis revealed a main effect of *facial attractiveness* (*F*_1,87_ = 228.72; *p* < 0.001, *η*_*p*_^2^ = 0.72) that was qualified by an interaction with *creativity* (*F*_1,87_ = 5.65; *p* = 0.02, *η*_*p*_^2^ = 0.06). A significant effect of *creativity* (*F*_1,87_ = 34.23; *p* < 0.001, *η*_*p*_^2^ = 0.28) was qualified by an interaction with *sex of participant* (*F*_1,87_ = 4.41; *p* = 0.04, *η*_*p*_^2^ = 0.05).

There was a significant two-way interaction between *face sex* and *participant sex* (*F*_1,87_ = 6.36; *p* = 0.013, *η*_*p*_^2^ = 0.07) and *face sex* and *creativity* (*F*_1,87_ = 54.38; *p* < 0.001, *η*_*p*_^2^ = 0.39). Consistent with hypotheses, a significant higher-order interaction was found between *face sex*, *facial attractiveness* and *creativity* (*F*_1,87_ = 5.94; *p* = 0.017, *η*_*p*_^2^ = 0.06). No other effects or interactions were significant (all *F* < 1.49, all *p* > 0.22).

**Figure 2.**
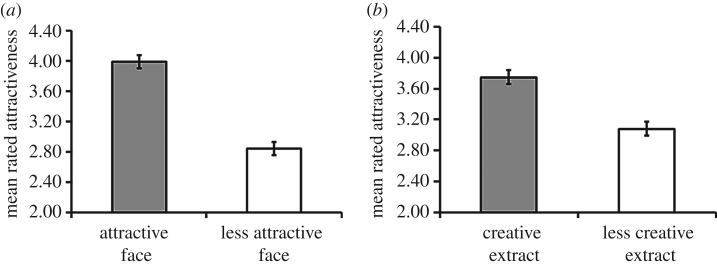
Independent effects of men's creativity and facial attractiveness on their overall attractiveness. The independent effects of facial attractiveness (*a*, Cohen's *d* = 1.36) and creativity (*b*, Cohen's *d* = 0.99) on men's attractiveness were both large.

To interpret the significant three-way interaction between *face sex*, *facial attractiveness* and *creativity*, separate 2 × 2 ANOVAs were conducted on the within-subjects factors *facial attractiveness* and *creativity*, for judgements of women's faces and judgements of men's faces. Analyses on judgements of men's faces revealed a significant effect of *creativity* (*F*_1,88_ = 87.51; *p* < 0.001, *η*_*p*_^2^ = 0.50), a significant effect of *facial attractiveness* (*F*_1,88_ = 163.40; *p* < 0.001, *η*_*p*_^2^ = 0.65) and no significant interaction between *creativity* and *facial attractiveness* (*F*_1,88_ = 0.03; *p* = 0.87). Paired samples *t*-tests collapsed across facial attractiveness revealed that men with creative story-telling ability (*M* = 3.75, s.e.m. = 0.09) were judged as more attractive than men with less creative story-telling ability (*M* = 3.08, s.e.m. = 0.09, *t*_89_ = 9.36; *p* < 0.001, *d* = 0.99). When collapsed across creative ability, men with attractive faces (*M* = 3.99, s.e.m. = 0.09) were judged as more attractive than men with less attractive faces (*M* = 2.84, s.e.m. = 0.10, *t*_89_ = 12.78; *p* < 0.001, *d* = 1.36, see figure 2).

**Figure 3.**
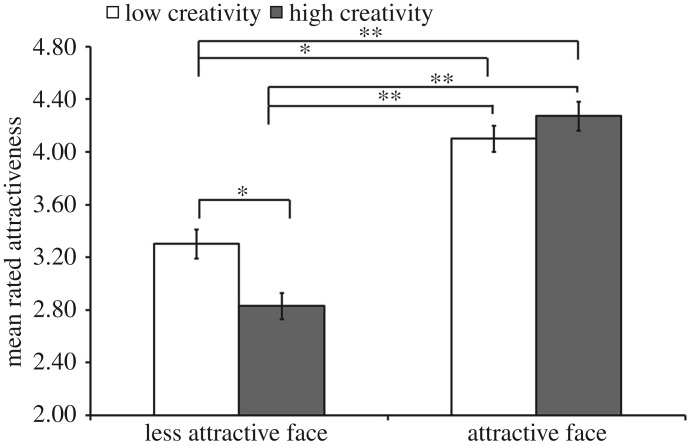
The significant two-way interaction between *facial attractiveness* and *creativity* in judgements of women's attractiveness (*η*_*p*_^2^ = 0.20). Asterisks indicate medium (*) and large effect sizes (**) using Cohen's *d*.

Data for judgements of women's faces revealed a significant effect of *creativity* (*F*_1,88_ = 5.07; *p* = 0.03, *η*_*p*_^2^ = 0.06), a significant effect of *facial attractiveness* (*F*_1,88_ = 186.02; *p* < 0.001, *η*_*p*_^2^ = 0.68) and a significant interaction between *creativity* and *facial attractiveness* (*F*_1,88_ = 22.15; *p* < 0.001, *η*_*p*_^2^ = 0.20, figure 3). This significant interaction reflected, in part, high creativity *reducing* the overall attractiveness of less attractive women (*t*_88_ = 4.90; *p* < 0.001, *d* = 0.52). Facially attractive and creative women were preferred relative to less creative, less attractive women (*t*_88_ = 8.12; *p* < 0.001, *d* = 0.86) and relative to creative women with less attractive faces (*t*_88_ = 14.54; *p* < 0.001, *d* = 1.54). Facial attractiveness enhanced the overall attractiveness of less creative women (*t*_88_ = 6.98; *p* < 0.001, *d* = 0.74) and less creative but attractive women were preferred relative to creative but less attractive women (*t*_88_ = 14.27; *p* < 0.001, *d* = 1.51). Creativity did not enhance the overall attractiveness of women with attractive faces (*t*_88_ = 1.85; *p* = 0.07, *d* = 0.20).

## 4. Follow-up experiment: replication using a measure of divergent thinking

As written creative expression and appreciation is, by definition, highly variable from the point of view of the reader and the writer, it is important to establish whether the current pattern of results generalize beyond the stimuli used in the current experiment (i.e. to creative thinking across different contexts). Thus, a follow­-up experiment was conducted using a well-established measure of divergent (i.e. creative) thinking (the alternative uses test; [29]; see e.g. [30] for recent discussion) in order to generate simple response lists that differ in creativity. Importantly, this replication attempt will reveal the extent to which the initial higher-order interaction between face sex, attractiveness and creativity generalizes across contexts, as response lists that differ in creativity remove aspects of semantic content that are not related to impressions of the producer's creativity (e.g. other impressions of character or intent inferred from idiosyncrasies within a short piece of creative writing).

## 5. Method

### 5.1. Participants

One hundred and twenty-five participants were recruited using the ‘buy responses’ function on surveymonkey.com. All participants were volunteers (donations to charities are offered as incentives in exchange for participation), and were selected from a wide panel of respondents via surveymonkey, with the experimenter selecting target criteria of an 18–30-year-old American sample to ensure a comparable mean age to the initial experiment. The experimenter specified a sample size of 90 and estimated response rate of 75–100% (i.e. surveymonkey send the task to more respondents based on this criteria). Participants were excluded from analyses if they did not respond to all trials in the experiment or did not specify a gender of male or female. One participant was also excluded for responding at one end of the response scale on every trial. This resulted in a final sample size for analysis of 104 participants (49 of whom were male, *M*_age_ = 24.51 years, s.d. = 5.07 years, three participants did not specify their age). Online and laboratory studies produce equivalent results (e.g. [31]; reviewed in [32]).

### 5.2. Face stimuli and creative responses

The follow-up experiment used identical face stimuli to the initial experiment. In order to generate a set of creative responses, a separate group of thirty-one volunteers (14 of whom were male, *M*_age_ = 24.29 years, s.d. = 6.94 years) completed a widely used measure of divergent thinking (the alternative uses task; [29]; Form B, part 2; see e.g. [30] for recent discussion) in class under test conditions. Following the user manual, participants had up to 4 min to think of up to six alternative uses for three everyday objects listed in the booklet. Responses to one object (a car tyre) were selected given a similar level of variability in the number of valid responses among males and females (*M*_male_ = 2.29 answers, *M*_female_ = 2.47 answers). To measure spontaneous creativity, and consistent with the instruction book, participants were not informed that the task measures creative thinking and were not encouraged to be creative in their responses. In line with the scoring guidelines, responses were selected if the use for the object was feasible and counted only if it differed from other responses or was unique from the example provided within the booklet. For both male and female participants, the number of valid responses to this question ranged from 0 to 6 alternative uses.

For participants who produced at least one valid response to this item (26 participants), all response lists were then rated by a separate panel of judges (three males and three females, *M*_age_ = 28.32 years, s.d. = 2.65 years) in an identical manner to the initial experiment (i.e. the same eight traits related to creativity and identical rating scale). Raters were informed that each list shows the response of one individual who was asked to list up to six alternative uses for the same everyday object (a car tyre), and that each individual was given the same amount of time, under test conditions, to do so (and to rate each extract in light of this). Agreement between raters was good (Cronbach's *α* = 0.86). Across raters, ratings of each list on all eight dimensions related to creativity were correlated with one another (all *r*_26_ > 0.63, all *p* < 0.01). Following the initial experiment, a composite measure of creativity was created by averaging all of the ratings for each list except its rated funniness (*M*_Global creativity_ = 4.01, s.d. = 1.61). From these ratings, eight male response lists (*M*_high male_ = 6.38, s.d. = 1.23, *M*_low male_ = 2.99, s.d. = 0.70) and eight female response lists (*M*_high female_ = 5.47, s.d. = 0.76, *M*_low female_ = 2.44, s.d. = 0.23) were selected. Half of the response lists were creative (e.g. ‘Use as a rope swing, cut in half and place side by side to make a Loch Ness Monster sculpture, as a garden planter, re-appropriate and use as parts for a go-kart, cut up and use as knee/elbow-pads’) and half were less creative (e.g. ‘Use as a seat’), with the two list sets differing from one another on global rated creativity excluding funniness (*t*_14_ = 7.53; *p* < 0.001). Within each set, lists did not differ in creativity according to the gender of the participant who produced the list (both *t*_6_ < 1.50, both *p* > 0.18). The same faces used in the same four conditions within the initial experiment were then randomly paired with lists by the experimenter, resulting in (separately for each gender) two attractive–creative face-list pairs, two attractive–less-creative face-list pairs, two less-attractive–creative face-list pairs and two less-attractive–less-creative face-list pairs. Lists and faces were matched by sex.

### 5.3. Procedure

The procedure, rating scale and processing of data in the follow-up experiment were identical to the initial experiment, except that participants were recruited to take part in a task rating the attractiveness of ‘thinkers’. Participants were informed that they would be asked to make judgements based on the responses of pictured individuals to a standard cognitive task used in psychology. They were informed that each of the 16 pictured individuals was provided with the same everyday object (a car tyre) and the same amount of time to list up to six alternative uses for that object under test conditions. They were asked, for each individual, to read that person's response to the task (presented below the face, with each answer presented in list form) and rate how attractive they judge each person to be.

## 6. Results

A mixed-design ANOVA with the within-subjects factors *face sex* (male and female), *facial attractiveness* (high and low) and *creativity* (high and low) and the between-subjects factor *sex of participant* (men and women) was conducted. This analysis revealed a main effect of *face sex* (*F*_1,102_ = 14.81; *p* < 0.001, *η*_*p*_^2^ = 0.13), a main effect of *creativity* (*F*_1,102_ = 27.42; *p* < 0.001, *η*_*p*_^2^ = 0.21), and a main effect of *facial attractiveness* (*F*_1,102_ = 171.78; *p* < 0.001, *η*_*p*_^2^ = 0.63). The main effect of *creativity* was qualified by an interaction with *sex of participant* (*F*_1,102_ = 4.23; *p* = 0.04, *η*_*p*_^2^ = 0.04) and was, separately, qualified by an interaction with *face sex* (*F*_1,102_ = 68.45; *p* < 0.001, *η*_*p*_^2^ = 0.40). The main effect of *facial attractiveness* was qualified by an interaction with *face sex* (*F*_1,102_ = 8.08; *p* < 0.01, *η*_*p*_^2^ = 0.07). Consistent with the initial experiment, a higher-order interaction was observed between *face sex*, *facial attractiveness* and *creativity* (*F*_1,102_ = 24.86; *p* < 0.001, *η*_*p*_^2^ = 0.20). No other effects or interactions were significant (all *F* < 1.25, all *p* > 0.26).

**Figure 4.**
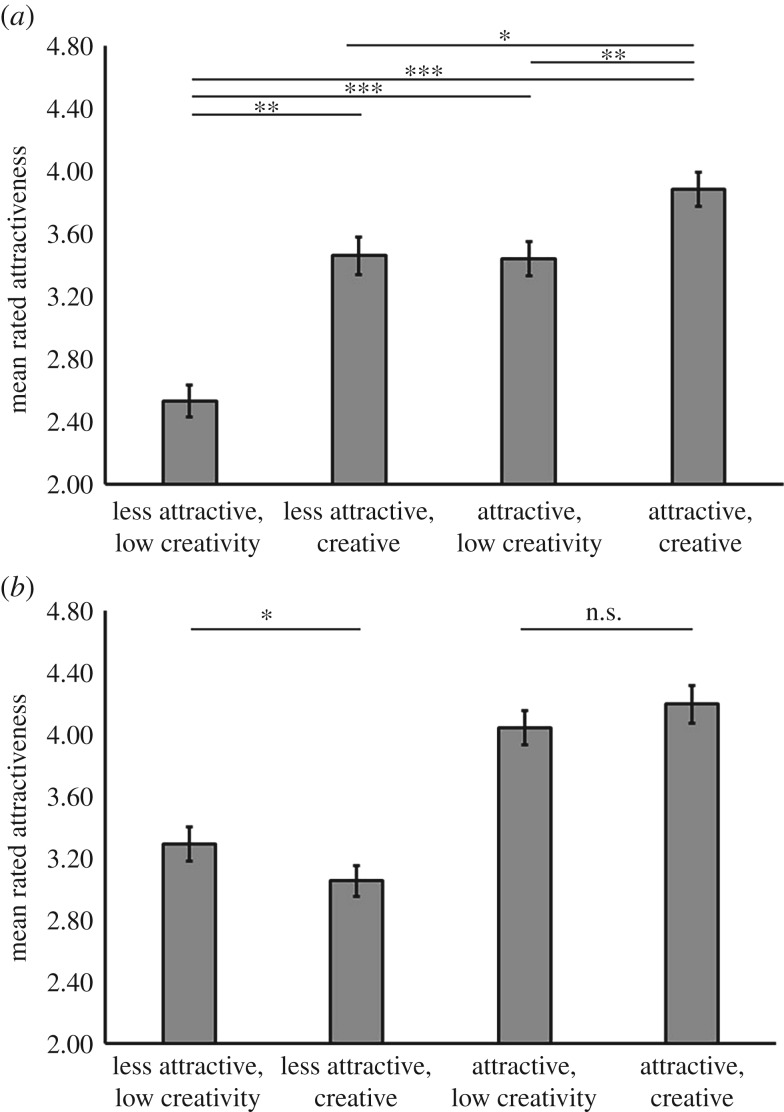
Replication of the significant interaction between face sex, creativity and facial attractiveness on overall attractiveness (*η*_*p*_^2^ = 0.20). (*a*) Creativity has a more substantial effect on the attractiveness of men with average faces than it does for men with attractive faces (asterisks indicate * small, ** medium and *** large effect sizes). (*b*) Facial attractiveness has a more substantial effect on women's attractiveness than does their creativity. When comparing conditions (panel *b* only), all differences (four comparisons) represent a large effect size (Cohen's *d*) unless otherwise indicated (two comparisons).

In order to interpret the significant three-way interaction between *face sex*, *facial attractiveness* and *creativity*, separate 2 × 2 ANOVAs were conducted on the within-subjects factors *facial attractiveness* and *creativity*, for judgements of women's faces and judgements of men's faces. Analyses on judgements of men's faces revealed a main effect of *creativity* (*F*_1,103_ = 71.34; *p* < 0.001, *η*_*p*_^2^ = 0.41), a main effect of *facial attractiveness* (*F*_1,103_ = 57.97; *p* < 0.001, *η*^2^*p* = 0.36) and, in contrast to the initial experiment, an interaction between *creativity* and *facial attractiveness* (*F*_1,103_ = 12.60; *p* < 0.01, *η*_*p*_^2^ = 0.11, figure 4*a*). Paired samples *t*-tests to interpret this interaction demonstrated that overall rated attractiveness differed among all four conditions in the task (all absolute *t* > 3.41, all *p* < 0.01, all 0.32 < *d* < 1.13), except for creative but less attractive men and attractive but less creative men, who were equivalent in overall attractiveness (*t*_103_ = 0.20; *p* = 0.84). Crucially, the interaction demonstrates that the positive effects of creativity on overall attractiveness are relatively more substantial for less attractive men (*d* = 0.75) than they are for attractive men (*d* = 0.50).

Analyses of judgements of women's faces revealed a main effect of *facial attractiveness* (*F*_1,103_ = 190.60; *p* < 0.001, *η*_*p*_^2^ = 0.65) which was qualified by an interaction with *creativity* (*F*_1,103_ = 10.62; *p* < 0.01, *η*_*p*_^2^ = 0.09). There was no main effect of *creativity* (*F*_1,103_ = 0.40; *p* = 0.53). Paired samples *t*-tests to interpret this interaction demonstrated that overall rated attractiveness differed among all conditions in the task (all absolute *t* > 2.63, all *p* < 0.01, all 0.25 < *d* < 1.15) except for less creative but attractive women and creative but attractive women, who were equivalent in overall attractiveness (absolute *t*_103_ = 1.69; *p* = 0.094, *d* = 0.17, figure 4*b*).

## 7. Final experiment: replication of initial experiment using a separate set of face stimuli

In order to establish whether the reported pattern of significant results and findings in the initial experiment generalize to other sets of faces, an additional online experiment was conducted using a new set of faces that had been rated for attractiveness by a separate panel of judges.

## 8. Method

### 8.1. Participants

Ninety-eight participants (60 males and 38 females, *M*_age_ = 24.13 years, s.d. = 3.17 years) were recruited via an online testing platform (prolific academic, www.prolific.ac see [33] for a recent review of this platform in behavioural research). All participants received the equivalent of £5 per hour for participating and were selected from a wide panel of potential respondents, with the experimenter selecting the target criteria of an 18–30-year-old sample. One participant was excluded from analysis for not specifying their gender.

### 8.2. Face stimuli, creative responses and procedure

The final experiment used identical short-story extracts to the initial experiment. A separate widely used database of face photographs [34] were used in this experiment. Eight male and eight female faces were selected from the full face set, with four attractive faces selected for each sex and four faces from around the mid-point of the entire set selected for each sex. The full set of photographs were of Caucasian individuals aged 18–29 taken in a standardized manner with neutral expression and direct gaze (639 × 480 pixels). Faces were standardized on pupil position and masked to remove clothing and background and to minimize hair cues. These faces were then rated in a randomized order in the centre of the screen by a separate panel of judges in an online study run via surveymonkey.com (male face ratings: *N* = 21 females, 12 males, *M*_age_ = 26.84 years, s.d. = 8.56 years; female face ratings: 35 males, 25 females, 2 undisclosed gender, *M*_age_ = 29.19 years, s.d. = 9.64 years) for attractiveness on a 1 (much less attractive than average) to 7 (much more attractive than average) scale. Participants were randomly allocated to rate either 36 women's faces or 36 men's faces. Scores for each face set were then standardized separately. The mean attractiveness rating of the attractive faces (*M*_men_ = 1.54, *M*_women_ = 1.55) was greater than the mean attractiveness rating of the less attractive faces (*M*_men_ = −0.06, *M*_women_ = −0.04, *t*_14_ = 19.42; *p* < 0.001). Within each face set, male and female faces did not differ from one another on rated attractiveness (both *t*_6_ < 0.24 both *p* > 0.82). As in the initial experiment, extracts were then randomly paired with faces by the experimenter and all participants viewed the same face-extract pairings. The procedure, rating scale and processing of data in this experiment were identical to the initial experiment.

## 9. Results

A mixed-design ANOVA with the within-subjects factors *face sex* (male and female), *facial attractiveness* (high and low) and *creativity* (high and low) and the between-subjects factor *sex of participant* (men and women) was conducted. This analysis revealed a main effect of *face sex* (*F*_1,96_ = 14.97; *p* < 0.001, *η*_*p*_^2^ = 0.14), a main effect of *facial attractiveness* (*F*_1,96_ = 75.56; *p* < 0.001, *η*_*p*_^2^ = 0.44) and a main effect of *creativity* (*F*_1,96_ = 12.74; *p* < 0.01, *η*_*p*_^2^ = 0.12). The main effect of *face sex* was qualified by an interaction with *facial attractiveness* (*F*_1,96_ = 6.66; *p* = 0.011, *η*_*p*_^2^ = 0.07). The main effect of *creativity* was qualified by an interaction with *facial attractiveness* (*F*_1,96_ = 7.85; *p* < 0.01, *η*_*p*_^2^ = 0.08). No other effects or higher-order interactions were significant (all *F* < 2.58, all *p* > 0.11). The interaction between *face sex* and *facial attractiveness* reflected a more substantial effect of *facial attractiveness* on women's overall attractiveness (*t*_97_ = 9.53; *p* < 0.001, *d* = 0.97) than on men's overall attractiveness (*t*_97_ = 4.73; *p* < 0.001, *d* = 0.48). The interaction between *creativity* and *facial attractiveness* reflected an effect of *creativity* on the overall attractiveness of less attractive faces (*M*_Creative_ = 3.81, s.e.m. = 0.11, *M*_Less Creative_ = 3.49, s.e.m. = 0.11, *t*_97_ = 4.08; *p* < 0.001, *d* = 0.41) but no effect of *creativity* on the overall attractiveness of attractive faces (*M*_Creative_ = 4.33, s.e.m. = 0.10, *M*_Less Creative_ = 4.31, s.e.m. = 0.10, *t*_97_ = 0.30; *p* = 0.77).

## 10. Discussion

The data provide evidence across two experiments that social knowledge of creativity shapes differences in attractiveness judgements of men compared with women, when integrated with knowledge of their facial attractiveness. The initial experiment demonstrated a positive effect of creativity on men's attractiveness, independent of their facial attractiveness, when creativity was measured in the form of written interpretations of a painting. Of note, these two independent effects of creativity and facial attractiveness on overall attractiveness were both large in size. In the follow-up experiment, using a well-established measure of creative/divergent thinking, although the positive effects of creativity were not independent from the positive effects of facial attractiveness when participants judged men, the interaction between creativity and facial attractiveness demonstrated that creativity had a more substantial effect on the attractiveness of men with less attractive faces than it did for men with attractive faces. Indeed, creative men with less attractive faces were equivalent in attractiveness to attractive, but less creative, men. In a final experiment, where the initial experiment was repeated with a different set of face stimuli, an interaction between facial attractiveness and creativity was observed which, this time, was not qualified by a higher-order interaction with the sex of face judged. Collectively, these findings suggest, across three experiments, that creativity may provide ‘leverage’ for less physically attractive individuals as social and/or romantic partners.

By contrast, for women, two of the three experiments demonstrated that facial attractiveness enhanced their overall attractiveness to a greater extent than creativity (written expression and creative thinking) enhanced their overall attractiveness. Indeed, across these experiments, creativity *weakened* the appeal of women with less attractive faces and did not benefit their attractiveness when displayed by women with attractive faces. This former unexpected finding (weakening attractiveness judgements) may suggest evidence for subtle denigration of creative women based on (low) physical attractiveness, although caution is urged in this interpretation in light of the findings of the final experiment, where creativity strengthened the attractiveness of both men and women with less attractive faces but did not enhance the attractiveness of men or women with more attractive faces. Collectively, the data provide novel evidence that creativity in the form of spontaneous written expression and creative ideas may have specific effects on men's attractiveness as a potential mate and/or social partner, although further independent replications (i) with novel face stimulus sets, (ii) across modalities (e.g. measuring indices of vocal rather than facial attractiveness) and (iii) other forms of creative expression could help to clarify the extent to which these findings generalize to responses to men compared to women.

While complementing work on the importance of cognitive intelligence and/or creativity for mate choice in humans (e.g. [9,11]) and non-human species (e.g. [1,2]), the findings develop recent theoretical proposals on the importance of novelty and innovation in information transmission between groups more generally [6–8]. The data for male creativity and attractiveness is consistent with the initial proposal that attraction to creativity may generalize to non-mating contexts as language transmits potentially useful information at no loss to the transmitter [22]. Moreover, indices of intelligence are thought to be important for group cooperation and cohesion [25], which, in turn, can facilitate access to resources and/or mates among males of other species (e.g. [26]). Indeed, as engagement with forms of art such as literary fiction improves performance on measures of social intelligence (e.g. theory of mind tasks; [35]), attraction to innovators may facilitate access to novel information in ways that increase the intellectual and social ‘leverage’ [24] of both male and female social groups.

These findings address an underexplored area of the literature, by testing for variation in appreciation of the creative output of men and women. While prior work demonstrates that creativity is attractive in both men and women and is enhanced in the light of motives to attract a potential mate [12], these experiments do not consider how knowledge of creativity is integrated with knowledge of physical appearance. Here, findings in two of three experiments suggest that creative output may (unconsciously) have greater net benefits on male than female success in mating competition, all else being equal. Moreover, they provide further evidence for the utility of testing for integration of social knowledge with surface cues in social judgements of others ([19]; see also [36]).

Recent reviews have highlighted the need to test whether simultaneous assessment of observable cues to attractiveness and intelligence shapes mate choice (reviewed in [1]). Similar paradigms to the current experiment may prove fruitful in testing for integration of knowledge of cognitive intelligence with knowledge of ‘quality’, in the assessment both of potential mates and/or allies. In humans, testing for equivalent effects of creativity in other domains (e.g. musicality) in tightly controlled experiments may also prove fruitful.

In conclusion, these findings present direct experimental evidence for a potential strategic advantage to creative displays in the form of written expression, which generalizes across contexts to creative thought more generally, and ‘offsets’ putative cues to low biological ‘quality’ in faces. Evidence for women across two of three experiments suggests that facial attractiveness enhances their overall attractiveness while creativity does not, which could potentially shape sex differences in creative output in response to social evaluation, while, across all experiments, the findings suggest that creative displays can strengthen the attractiveness of individuals both as social and romantic partners.

## Supplementary Material

Data file for Study 1

## Supplementary Material

Data file for Study 2

## Supplementary Material

Data file for Study 3

## Supplementary Material

Codebook

## Data accessibility

All data files and associated codebook are contained as part of the electronic supplementary material.
